# A Phytochemical-Based Copolymer Derived from *Coriolus versicolor* Polysaccharopeptides for Gene Delivery

**DOI:** 10.3390/molecules23092273

**Published:** 2018-09-06

**Authors:** Wing-Fu Lai, Marie C. Lin, Guping Tang

**Affiliations:** 1 Department of Applied Biology and Chemical Technology, The Hong Kong Polytechnic University, Kowloon, Hong Kong, China; 2 School of Pharmaceutical Sciences, Health Science Center, Shenzhen University, Shenzhen 518060, China; 3 Academy of Medical Science, Zhengzhou University, Zhengzhou 450001, China; mcmlin@163.com; 4 Department of Chemistry, Zhejiang University, Hangzhou 310058, China

**Keywords:** *Coriolus versicolor*, herbs, gene delivery, poly(ethylenimine), polysaccharopeptide

## Abstract

*Coriolus versicolor* is an herb widely used for cancer treatment in traditional Chinese medicine. Its active ingredients, polysaccharopeptides (PSP), have been used for adjuvant therapies in cancer treatment. This study conjugates *Coriolus versicolor* PSP with poly(ethylenimine) (PEI) to generate a PSP-PEI copolymer for gene transfer. After PEI conjugation, both the pH buffering capacity and DNA compaction ability of PSP are significantly increased. Compared with that of PSP, the transfection efficiency of PSP-PEI is 10 to 20-fold higher in vitro. This is a proof-of-concept study reporting the direct use of bioactive phytochemicals from traditional Chinese medicine for gene vector development. The promising performance of PSP-PEI raises the possibility that bioactive herbal ingredients can be further developed as a multi-therapeutic gene carrier for tackling cancers.

## 1. Introduction

*Coriolus versicolor* has a long history of application in oriental medicine. This fungus, which is also known as “turkey tails”, is an obligate aerobe commonly found on tree trunks, branches, dead logs, and stumps [1]. It has multiple botanical names such as *Boletus versicolor*, *Polyporus versicolor*, *Polystictus versicolor*, and *Trametes versicolor* [1]. According to *Compendium of Materia Medica* written during the Ming dynasty of China, there are more than 120 strains of *C. versicolor* [2]. Due to its unique antitumor effect, *C. versicolor* has emerged as one of the most important herbs used to tackle cancers. The antitumor properties of *C. versicolor* largely come from polysaccharopeptides (PSP). Studies have revealed that PSP can inhibit the proliferation of various cancer cells partly by activating immune cells, increasing the expression of cytokines and chemokines, and enhancing dendritic cell and T-cell infiltration into tumors [3]. PSP pre-treatment has also been found to increase the response of leukemia cells to apoptosis induced by camptothecin [4]. Since 1997, clinical trials on *C. versicolor* PSP have been performed in the mainland China. Results have shown that *C. versicolor* PSP can ameliorate side effects (such as pain, loss of appetite, vomiting, nausea and fatigue) caused by cancer treatment, and hence can improve patients’ quality of life [3]. Regarding the therapeutic potential of PSP as mentioned above, this study modifies PSP using low-molecular-weight (LMW) poly(ethylenimine) (PEI), which is one of the extensively studied polycations for non-viral gene delivery [5,6], to form a PSP-PEI copolymer as a gene carrier. After PEI conjugation, the pH buffering capacity, transfection efficiency and DNA compaction ability of PSP are significantly enhanced. Our results raise the possibility that herbal ingredients can be exploited as candidates for development of multi-therapeutic gene carriers for tackling cancers.

## 2. Results and Discussion 

### 2.1. Structural Modification of PSP

*C. versicolor* PSP are water-soluble substances having a dark brown colour and characteristic odour. Their polysaccharide moieties are highly complex, consisting of glucose molecules linked with different sugar units (e.g., galactose, mannose, arabinose, and xylose); whereas the peptide moieties contain a large amount of aspartic acid and glutamic acid, with acidic and neutral amino acids (such as leucine, glycine, alanine, threonine, serine, glutamic acid, valine and aspartic acid) accounting for 70% of all kinds of amino acids present [1]. As the physical and chemical properties of PSP have already been documented in the literature [1,3,4,7], readers are referred to those articles for details. In this study, we conjugate this extensively-studied phytochemical, which has been reported to exhibit anti-cancer properties [1], with PEI to generate a bioactive vector for cell transfection. PEI is a cationic aziridine polymer that exists as a polycation showing high pH buffering capacity over a broad range of pH values [8]. Previous studies have revealed that the transfection efficiency and cytotoxicity of PEI are positively related to the molecular weight of PEI [9,10,11]. As the aim of PEI incorporation in this study is to enhance the positive charge density of PSP, LMW PEI (e.g., PEI 0.8K) is adopted because it can serve the purpose and is less toxic than its high-molecular-weight counterparts.

**Figure 1:**
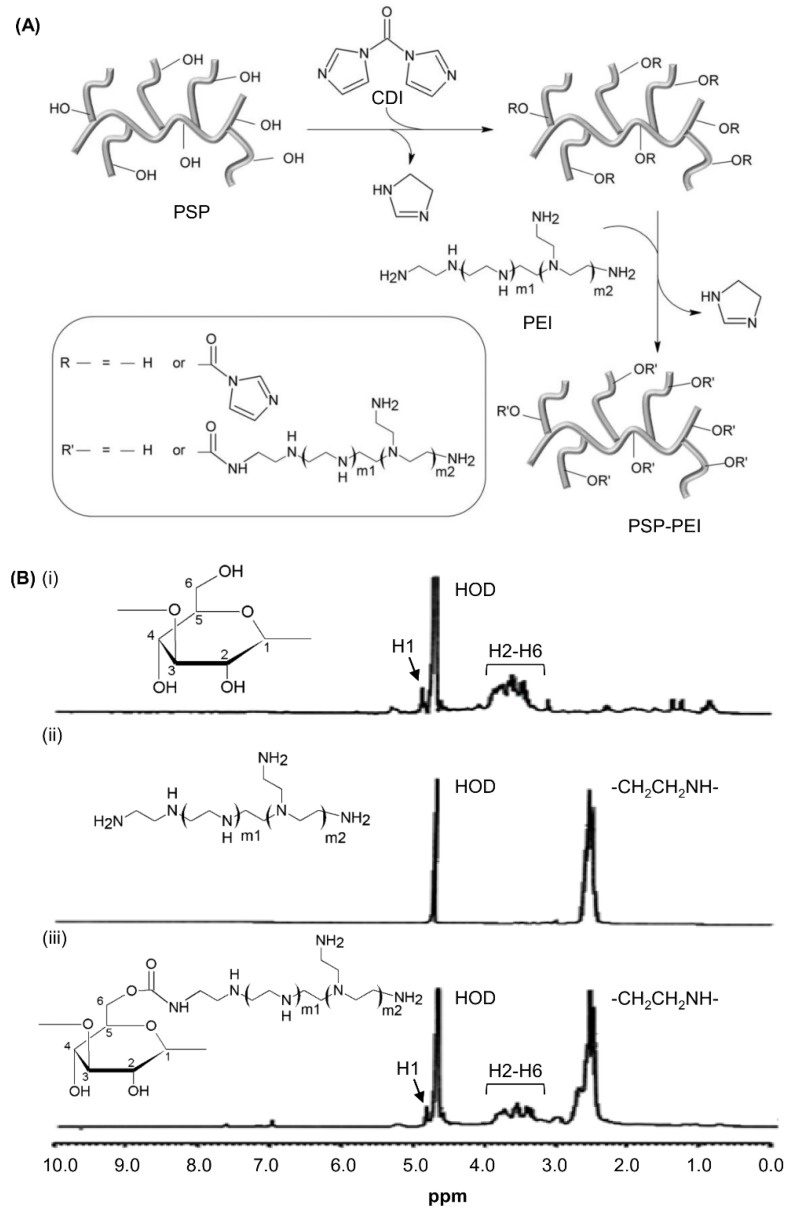
(**A**) Schematic diagram showing the steps involved in the synthesis of PSP-PEI. (**B**) ^1^H-NMR spectra of (i) PSP, (ii) PEI, and (iii) PSP-PEI in D_2_O.

The synthetic route of PSP-PEI is shown in Figure 1A. During synthesis, 1,1′-carbonyldiimidazole (CDI), which activates the hydroxyl groups of PSP to form active imidazolyl carbamate intermediates, is used as a coupling agent. The intermediates are subsequently attacked by the primary amine groups of PEI to form PSP-PEI, with imidazole being released as a byproduct. The structures of PSP, PEI and PSP-PEI were elucidated by proton nuclear magnetic resonance (^1^H-NMR) (Figure 1B). In the spectrum of PSP, the signal from the anomeric proton of the glucopyranosyl ring (5.15 ppm) is well separated from other proton signals (3.7–4.3 ppm). The low-intensity signals at 5.2 ppm and 5.3 ppm are attributed to the protons at the anomeric carbon of the α-1,3 and α-1,4 glucosidic linkages, respectively. The proton resonance signals at 0.7–2.5 ppm represent the amino acid side chains in PSP. All these peaks are found in the spectrum of PSP-PEI but not in the spectrum of PEI. This, together with the appearance of PEI CH_2_ signals resonating between 2.5 and 2.7 ppm in the spectrum of PSP-PEI, suggests successful PEI conjugation to PSP. The molar ratio of PSP to PEI in PSP-PEI is calculated based on the proton integral values at 5.15 ppm (C-1 hydrogen of PSP) and 2.5–2.7 ppm (CH_2_ of PEI) in the spectrum of PSP-PEI. It is approximated to be around 1:100 to 1:200.

### 2.2. Polyplex Formation and Characterization

**Figure 2:**
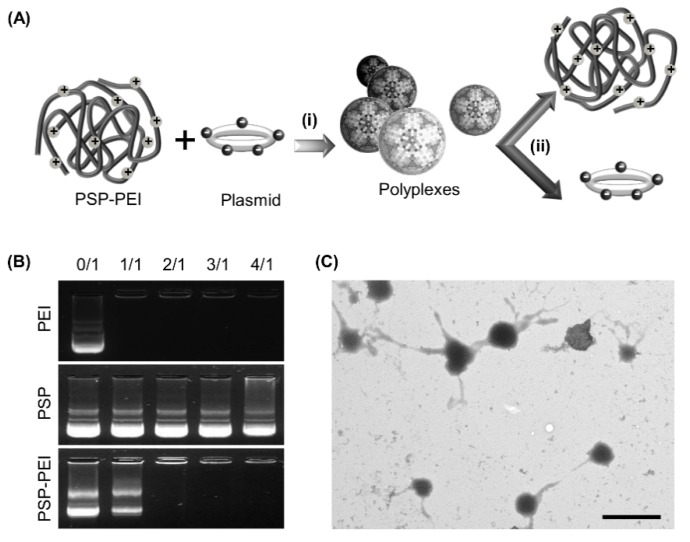
(**A**) Schematic diagram showing (i) the formation of polyplexes via electrostatic interactions between DNA and PSP-PEI, and (ii) the subsequent dissociation process undertaken by the polyplexes after cellular internalization. (**B**) Gel retardation assay of polyplexes prepared with different polymers (PEI 25kDa, PSP and PSP-PEI) at various polymer/DNA mass-to-mass ratios: 0/1, 1/1, 2/1, 3/1, and 4/1. (**C**) A representative TEM micrograph of polyplexes formed between PSP-PEI and DNA. The scale bar is 1 μm.

Polyplexes are formed between PSP-PEI and DNA through complex coacervation (Figure 2A). Although there is, as yet, no generally accepted theory of complex coacervation [12], it is thought that electrostatic interactions between the oppositely charged polyions play a predominant role [13,14,15]. Upon incorporating PEI into PSP, the presence of the positively charged PEI amine groups enables PSP to effectively aggregate with DNA, which is negatively charged due to the presence of phosphate groups, by electrostatic interactions. This mechanism of DNA condensation is needed for transporting DNA through the plasma membrane during gene delivery [14]. The success of DNA condensation mediated by PSP-PEI is evidenced by the results of the gel retardation assay. As shown in Figure 2B, no DNA retardation can be observed for unmodified PSP, even up to a polymer/DNA mass-to-mass ratio of 4/1. But after PEI conjugation, the DNA condensing ability of PSP is significantly improved, and is comparable to that achieved by PEI 25 kDa, which can retard DNA completely at a polymer/DNA mass-to-mass ratio of 1/1.

Formation of polyplexs between PSP-PEI and DNA is further examined by transmission electron microscopy (TEM). A micrograph of the polyplexes is shown in Figure 2C. Polyplex formation is mediated by electrostatic interactions between PSP-PEI and DNA. Such interactions may produce aggregates with low configurational entropy, as suggested by the “dilute phase aggregate model” [16]. The coacervate phase can be formed upon rearrangement in the system. The rearrangement process is proposed to be driven by a gain in configurational entropy [16], which occurs when the polyion mixture is separated into two distinct phases [17]. One is the dilute equilibrium phase. The other is the randomly mixed, dense coacervate phase with a relatively high concentration of the polyions [13].

### 2.3. Efficiency of PSP-PEI in Transfection

**Figure 3:**
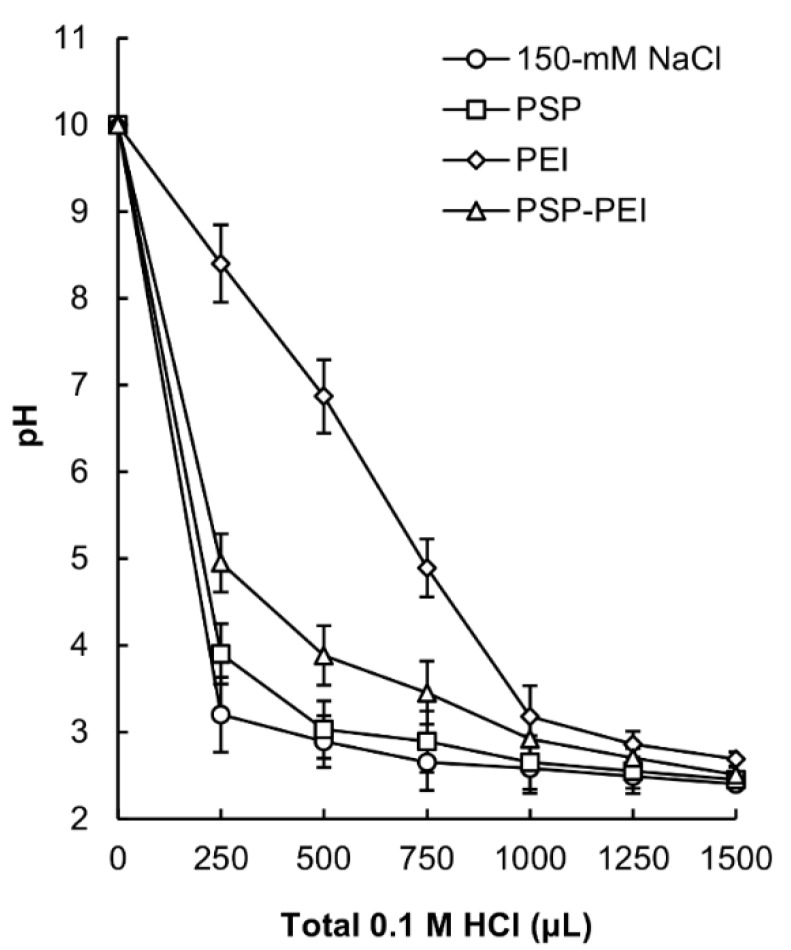
Acid-base titration profiles of PEI 25 kDa, PSP and PSP-PEI in a 150 mM NaCl solution.

The pH buffering capacity of PSP is increased after PEI conjugation, as shown in Figure 3. This is attributed to the presence of PEI amine groups. The pH buffering capacity may reflect the ability of a polymer to enhance osmotic swelling and rupture of endosomes via proton sponge effects upon cellular internalization [9,18,19,20]. Taking this into account, the higher buffering capacity of PSP-PEI as compared to that of PSP suggests that PSP-PEI may disrupt the vesicle membrane more effectively to release polyplexes into the cytoplasm and hence may facilitate the process of cell transfection.

The higher transfection efficiency of PSP after PEI conjugation is demonstrated by the EGFP transfection efficiency assay in AGS and U87 cells. Cancer cell lines are adopted in this study so as to evaluate the possibility of using PSP, after PEI modification, as a gene carrier for future gene therapy, on top of the possible pharmaceutical interventions mediated by PSP per se.

**Figure 4:**
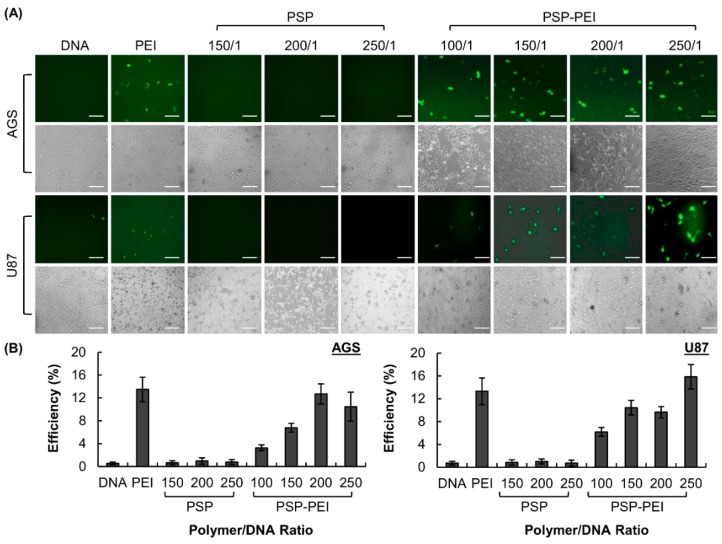
(**A**) Representative EGFP fluorescence images and phase-contrast images of AGS and U87 cells transfected with PEI 25 kDa, PSP, and PSP-PEI at different polymer/DNA mass-to-mass ratios. The polymer/DNA ratio adopted for PEI 25 kDa is 2/1. The scale bar is 200 μm. (**B**) The percentages of AGS and U87 cells expressing the EGFP protein after transfection with different polymers (PEI 25 kDa, PSP, and PSP-PEI) at various polymer/DNA mass-to-mass ratios. The polymer/DNA ratio adopted for PEI 25 kDa is 2/1.

As shown in Figure 4, AGS and U87 cells transfected with PSP-PEI give significant EGFP fluorescence. At the optimal polymer/DNA mass-to-mass ratio, the transfection efficiency obtained by PSP-PEI in AGS and U87 cells is 10 to 20-fold higher than that achieved by PSP or by DNA alone, and is comparable to that attained by PEI 25 kDa, which is used as a positive control. 

**Figure 5:**
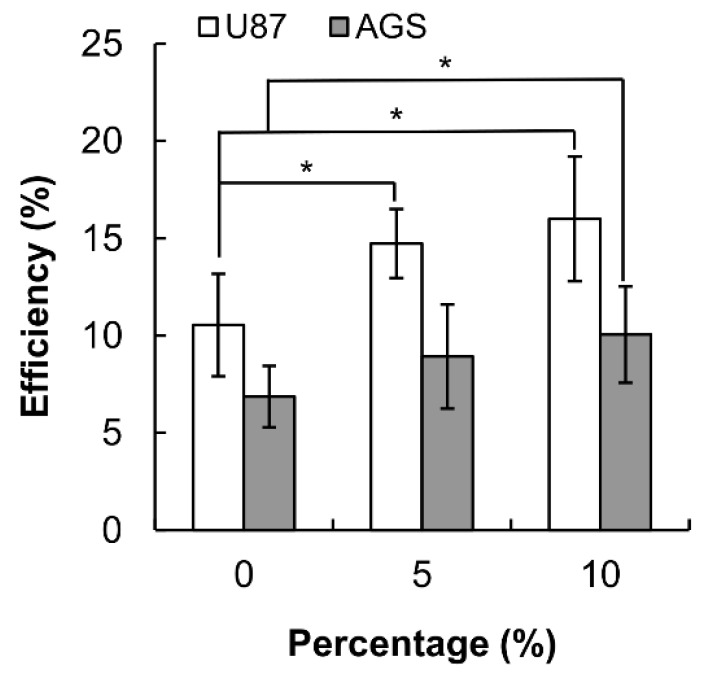
The percentages of AGS and U87 cells expressing the EGFP protein after transfection with PSP-PEI (at the polymer/DNA mass-to-mass ratio of 250/1) in different concentrations of FBS. (* *p* < 0.05; *n* = 5).

Furthermore, in the presence of fetal bovine serum (FBS), there is an increase in the transfection efficiency obtained by PSP-PEI. This may be because of the adsorption of serum proteins onto the polyplexes, thereby reducing the cytotoxicity of the polyplexes and thus enhancing the efficiency of transgene expression (Figure 5). The high efficiency of cell transfection, together with the well-documented anti-cancer and immunoregulatory properties of PSP [3,7,21,22,23,24], substantiates the potential use of the copolymer in medicinal and nucleic acid therapies.

### 2.4. Cytotoxicity of PSP-PEI

**Figure 6:**
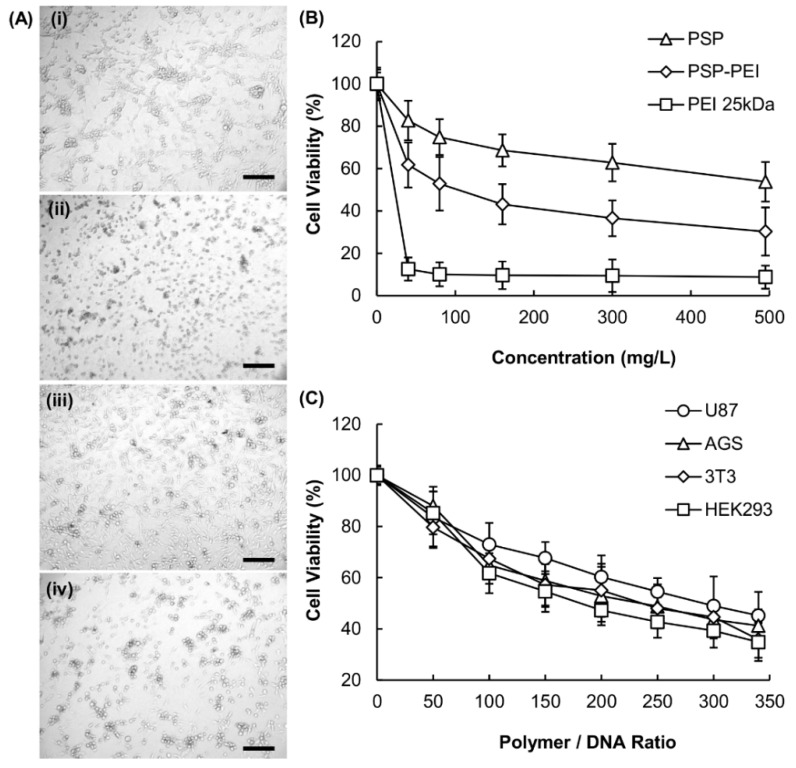
(**A**) Morphologies of U87 cells after transfection with (i) DNA alone, or with polyplexes generated from (ii) PEI 25 kDa, (iii) PSP, and (iv) PSP-PEI. The polymer/DNA ratios adopted for PEI 25 kDa, PSP, and PSP-PEI are 2/1, 250/1, and 250/1, respectively. The scale bar is 200 μm. (**B**) Viability of U87 cells treated with different concentrations of PEI 25 kDa, PSP, and PSP-PEI. (**C**) Viability of different cell lines treated with polyplexes formed between PSP-PEI and DNA at different polymer/DNA mass-to-mass ratios.

To determine the clinical applicability of a gene carrier, toxicity is a major factor to be considered. Based on microscopic evaluation, cells transfected with PEI 25 kDa show substantial morphological changes and experience cell lysis (Figure 6A). The presence of cell debris, as well as detachment from the bottom, is also observed. On the contrary, after transfection with PSP or PSP-PEI, no significant cell damage is found. The cytotoxicity of PSP-PEI is further examined using the MTT assay (Figure 6B,C), which suggests that the cytotoxic effects imposed by the copolymer are dependent on both the polymer/DNA mass-to-mass ratio adopted and the concentration of the copolymer. The cytotoxicity of PSP-PEI is thought to come largely from the PEI moiety because PSP is expected to have a high safety profile. The latter has been reported by an earlier study, which has reported that the highest daily tolerant dose of PSP for mice is as high as 18–20 g/kg [25]. Clinically, PSP has also been found to be nontoxic to patients even the dosage is several times higher than the therapeutic one [26].

## 3. Materials and Methods

### 3.1. Materials

PEI (0.8 kDa and 25 kDa), 3-(4,5-dimethylthiazol-2-yl)-2,5-diphenyltetrazolium bromide (MTT), CDI, triethylamine and dimethyl sulfoxide (DMSO) were purchased from Sigma-Aldrich (St. Louis, MO, USA). The highly concentrated PSP-containing essence of *C. versicolor* was purchased from U-Century (HK) Limited (Hong Kong SAR, China). All reagents were used as received unless otherwise specified.

### 3.2. Plasmid Preparation

The plasmid, pEGFP-N1 (BD Biosciences, San Jose, CA, USA), was amplified in DH5α *Escherichia coli* (Invitrogen; Carlsbad, CA, USA), followed by extraction with the Plasmid Giga Kit in accordance to the manufacturer’s protocols. The extracted plasmid was dissolved in Tris/EDTA buffer (1 μg/μL), and was stored at −20 °C for subsequent use.

### 3.3. Synthesis of PSP-PEI

PSP-containing essence (3.5 g) was suspended in distilled water (50 mL). The suspension was heated at 65 °C for 15 min, and was centrifuged at a relative centrifugal force of 2500× *g* for 10 min. The supernatant was filtered by microfiltration, followed by lyophilisation to obtain PSP powder. The powder (300 mg) was dissolved in distilled water (5 mL). Degassed DMSO (10 mL) and triethylamine (200 μL) were then added, followed by the addition of a DMSO solution (5 mL) containing CDI (0.1 g/mL). After 3 h of reaction, PEI (Mw = 0.8 kDa) in DMSO (0.14 g/mL) was added dropwise. The reaction mixture was left at ambient conditions for 24 h, and then was dialyzed against distilled water for 3 days with a molecular weight cut-off (MWCO) of 12 kDa before lyophilisation.

### 3.4. ^1^H-NMR

The structures of PSP, PEI and PSP-PEI were analysed by ^1^H-NMR using a Bruker 300 MHz NMR spectrometer (Bruker Analytic GmbH, Germany). 20 mg of the polymer were dissolved in 0.7 mL of deuterium oxide (D_2_O). The ^1^H-NMR spectra were obtained with 32 scans under ambient conditions.

### 3.5. Acid-Base Titration

PSP, PEI 25 kDa and PSP-PEI were evaluated for their pH buffering capacity as previously described [5].

### 3.6. Polyplex Formation and Microscopic Evaluation

Polyplexes were prepared by adding an aqueous solution of PSP or PSP-PEI into an equivalent volume of a plasmid solution (in Tris/EDTA buffer) at an appropriate polymer/DNA mass-to-mass ratio. The mixture was vortexed gently for 5 s, followed by incubation at ambient conditions for 15 min. The same procedure was adopted to generate polyplexes from PEI 25 kDa, with the polymer/DNA ratio being set to be 2/1. Polyplexes of PSP-PEI were imaged by TEM. In brief, a drop of the polyplex solution containing 0.1 wt% phosphotungstic acid was placed on a 200-mesh carbon-coated copper grid. After evaporation of the solvent at ambient conditions for 10 min, an image was taken using a transmission electron microscope (JEM-2010, JEOL, Tokyo, Japan) operating at an acceleration voltage of 100 kV.

### 3.7. Gel Retardation Assay

PSP, PEI and PSP-PEI were evaluated for their DNA condensation ability using the gel retardation assay. After formation of polyplexes, polyplex solutions with different concentrations were added into the wells of a 1% (*w/v*) agarose gel pre-stained with SYBR Safe DNA gel stain (Invitrogen, Carlsbad, CA, USA). Electrophoresis was then performed at 100 V for 40 min. The DNA bands were visualized under a blue-light transilluminator (Safe Imager™ 2.0 Blue-Light transilluminator, Invitrogen, Carlsbad, CA, USA).

### 3.8. Cell Culture

Human gastric adenocarcinoma AGS and human glioblastoma U87 cells were cultured in RPMI 1640 and MEM, respectively. 3T3 mouse fibroblasts and HEK293 cells were cultured in DMEM. All cell lines were obtained from ATCC (Manassas, VA, USA). All media were supplemented with 100 units/mL penicillin, 100 μg/mL streptomycin and 10% FBS before use. In the case of DMEM, l-glutamine was also added to reach a concentration of 2 mM.

### 3.9. Transfection Efficiency Assay

50,000 cells were seeded per well in a 24-well plate one day before transfection. During transfection, the growth medium in each well was replaced with 1 mL of the fresh cell culture medium containing 0%, 5%, or 10% of FBS. 300 µL of OPTI-MEM (containing polyplexes generated from 1 μg of pEGFP-N1 at an appropriate polymer/DNA mass-to-mass ratio) were added into each well, followed by 5-h incubation at 37 °C in 5% CO_2_. After that, the medium was aspirated and replaced with the fresh cell culture medium. After 48 h of post-transfection incubation, the percentage of fluorescence-positive cells was monitored by averaging the cell counts of five randomly chosen views under the fluorescence microscope as previously reported [6]. The experiment was replicated three times.

### 3.10. Cytotoxicity Assay

1,000 cells were seeded per well in a 96-well plate, and were incubated at 37 °C until a confluence of 70–80% was obtained. 50 µL of either the polyplex solution (containing 0.17 μg of pEGFP-N1) or the polymer solution were added to each well. The plate was then incubated at 37 °C for 5 h. After that, the solution was aspirated and replaced with the fresh cell culture medium. After 48 h of post-treatment incubation, the morphologies of the cells were observed under an optical microscope. In addition, 20 µL of the filtered MTT reagent (0.5 mg/mL) were added to each well. The unreacted reagent was removed by aspiration after 4 h. The violet crystals in each well were dissolved in 100 µL of DMSO. The colour intensity was measured by an ELISA reader at a wavelength of 595 nm. Cell viability (%) was calculated by the following formula:

(1) $$\mathrm{Cell}\;\mathrm{viability}\;\left(\%\right)=\left(\frac{{\left[A\right]}_{\mathrm{Test}}}{{\left[A\right]}_{\mathrm{Ctrl}}}\right)\times 100$$

where [A]_Test_ and [A]_Ctrl_ represent the ELISA readings for the tested well and control well, respectively.

### 3.11. Statistical Analysis

All data were expressed as the means ± standard deviation. Student’s unpaired *t*-test was performed to assess the statistical significance. Differences with a *p*-value < 0.05 were considered to be statistically significant.

## 4. Conclusions

*C. versicolor* is one of the medicinal fungi commonly used in traditional Chinese medicine. Earlier studies have documented that *C. versicolor* PSP display diverse therapeutic activities, from inhibition of tumor cell proliferation to immunomodulation [7,27,28,29]. Regarding the therapeutic potential of PSP, this study modifies PSP with PEI to generate a copolymer as a gene carrier. For future research, further evaluation of the bioactivity of PSP after PEI modification, particularly in the *in vivo* context, is needed before PSP-PEI can be applied practically to drug and gene therapies. More detailed characterization on the physical and chemical properties of the copolymer is also required during structural optimization of the copolymer. Nevertheless, results in this study have already supported the possibility of using PSP-PEI in gene delivery. Along with the documented therapeutic properties of PSP [1,3,29], PSP-PEI is a promising candidate to be further developed as a bioactive gene carrier for multimodal therapies.
